# Integrating immunisation services into nutrition sites to improve immunisation status of internally displaced persons’ children living in Bentiu protection of civilian site, South Sudan

**DOI:** 10.11604/pamj.2019.32.28.15464

**Published:** 2019-01-16

**Authors:** Olusola Oladeji, Penelope Campbell, Chandrakala Jaiswal, Dick Chamla, Bibilola Oladeji, Christopher Otti Ajumara, Lydie Maoungou Minguiel, Joseph Senesie

**Affiliations:** 1 Health section, UNICEF, Juba Country Office, South Sudan; 2 Nutrition section, UNICEF Bentiu Field Office, South Sudan; 3 Emergency Response Team, UNICEF, New York; 4 College of Medicine, University of Ibadan, Nigeria; 5 Health section, UNICEF Bentiu Field Office, South Sudan; 6 Nutrition section, UNICEF, Juba Country Office, South Sudan

**Keywords:** Integration, nutrition, immunization, OTP, PHCC, conflict

## Abstract

**Introduction:**

The protracted war in South Sudan has led to severe humanitarian crisis with high level of malnutrition and disruption of the health systems with continuous displacement of the population and low immunization coverage predisposing the population to vaccine preventable diseases. The study aimed at evaluating the effect of integrating immunization services with already established nutrition services on immunization coverage in resource-constrained humanitarian response.

**Methods:**

A community and health facility based interventional study involving integration of immunization into nutrition services in two Outpatient Therapeutic Program(OTP)centers in Bentiu PoC between January-December 2017. The main hypothesis was that inclusion of immunization services during nutrition services both at the OTP and community outreaches be an effective strategy for reducing missed opportunity for immunizing all eligible children accessing nutrition services. Data analyzed using STATA version 15 and bivariate analysis using logistic regression was conducted to identify predictor of missed vaccinations.

**Results:**

Integration of immunization into the nutrition services through the OTP centres increased the number of children immunized with various antigens and the dropout rate was much lower and statistically significant among children who received immunization at the OTP centers than those in the Primary Health Care Centers (PHC Centers) in the study sites. Children who were vaccinated at the OTP centre in sector 2 were 45% less likely to miss vaccination than those vaccinated at the PHCC (OR: 0.45; 95%CI:0.36- 0.55), p<0.05 while those vaccinated at the OTP sector in sector 5 were 27% less likely to miss vaccination than those vaccinated at the PHCC (OR: 0.27; 95%CI: 0.20 -0.35) p<0.05).

**Conclusion:**

This study indicated that immunization coverage improved effectively with integration with nutrition services as a model of an integrated immunization programme for child health in line with the Integrated Management of Childhood Illnesses (IMCI) and the Global Immunization Vision and Strategy (GIV).

## Introduction

Expanded Program on Immunization (EPI) has become one of the most successful public health programmes, reaching over 85% of the world's children [1]. However, EPI coverage has been plateauing the last 10 years and having not reached the set coverage targets; twenty-two million children, mostly living in the world's poorest countries who haven't received the full course of recommended vaccine by the age of 1 year [1] and integrated and comprehensive service delivery has been reported to have potential to generate demand, strengthen routine immunization services, reduce missed opportunities and improve coverage [2]. The protracted war in South Sudan has led to disruption of child survival interventions in all the counties with continuous displacement of the population as a result of the conflict. The coverage for all vaccine preventable diseases in South Sudan remains below 50 per cent, and is less than 20 per cent in the conflict affected Greater Upper Nile Region of Jonglei, Upper Nile and Unity states. The consistently low coverage predisposes returnees, refugees, IDPs, and the surrounding host community to the risk of infectious disease outbreaks given the increasing pool of unimmunized susceptible children as well as the poor living conditions with some of the counties reporting bouts of measles outbreaks almost every year especially among the IDPs due to poor immunization coverage [3]. In the Bentiu PoC, measles vaccination coverage verified by immunization card was found to be 23.4% during SMART survey conducted in 2016 and there was also reported outbreak of measles in 2016 as a result of significant unimmunized children with missed opportunities during provision of nutrition services in the camp [4]. A child who is borderline nourished will tip into malnutrition if he or she contracts an infectious disease such as measles. As part of an integrated health approach, immunization plays a critical role in curbing the devastating impact of malnutrition.

WHO and UNICEF developed the Global Immunization Vision and Strategy (GIVS), in 2005, to expand the reach of EPI, and prevent more disease [5]. In May 2012, the Global Vaccine Action Plan (GVAP) framework was endorsed at the World Health Assembly (WHA) to achieve the Decade of Vaccines' vision of delivering universal access to immunizations [1]. One of the six GVAP principles is integration, stating: “strong immunization systems, as part of broader health systems and closely coordinated with other primary health care delivery programmes, are essential for achieving immunization goals.” This promotes a strong immunization system as an integral part of a well-functioning health system, as well as the development of appropriate interventions for integration, to maximize the synergistic effects [1, 5]. However, various studies have suggested that the design of integrated outreach services should be informed by local experience supplemented by lessons learned elsewhere [6-8]. In most countries, there are more service delivery points for immunization than other services like nutrition and in such settings, immunization platforms are being used to improve access to non-vaccine programs [1]. However, in South Sudan similar to what obtains in other low-income countries, lack of regular or adequate funding has been commonly cited for poor implementation of immunization outreach activities. For example, cost and financing assessment for Ethiopia's National Immunization Program [9] found that operational costs (primarily transport and per diem payments) for integrated outreach were consistently underfunded or not funded at all. In response to the food crises being reported in most of the conflict affected states in South Sudan including the study site, significant fund has been deployed to address the malnutrition associated with the food crises and so better resources are available for nutrition platform to be used in improving immunization services especially community outreaches. In addition, the Ready to use Therapeutic Food (RUTF) being provided to the malnourished children at the Out Patient Therapeutic Program (OTP) centers serve as a form of incentives for mother and care givers to travel long distance when needed to access the nutrition services much more than they do to receive immunization services for their children This study aimed at evaluating the effect of integrating immunization services in the nutrition services on immunization coverage in a resource constrained humanitarian response.

**Justification for the study:** The OTP centers are located few meters to the PHC centers where immunizations are being conducted and mothers were usually asked to go to the PHCC after being attended to at the OTP centers for their children immunization. However, it was observed that most of the children referred from the OTP centers to the PHCC for immunization services didn't go. During a focus group discussion conducted among the mothers/care givers some of the reasons given by the mothers/care givers for not taking their children for immunization at the PHCC when referred from OTP centers included being tired after spending some considerable time at the OTP centre and the need to go back home to continue their work or household chores. Some caregivers reported that on few occasions they went, the vaccinators were not around to provide the services or being asked to queue up again on getting to the clinic instead of being attended to immediately while others reported they forgot the immunization cards and had to go back home but late to go back to the clinic for that day leading to missed opportunity to improve immunization coverage in the camp.

## Methods

This was a community and health facility based interventional study during which immunization services were integrated with nutrition services in OTP centres and nutrition outreaches where immunization was previously not provided. All children under 5 years seen at the OTP centres and during nutrition community outreaches were all assessed for their immunization status and provided with appropriate immunization needed during the course of the study between January-December 2017. In the 2 OTP centers where the intervention was implemented, vaccinators were recruited to join the nutrition team to provide immunization in the OTP centre. During community outreaches and at the OTP centers the client flow as arranged so that all children who visited the centre either on follow up visit or new cases or during mass nutrition screening conducted by the Community Nutrition Volunteers were first assessed for immunization status before being screened or and enrolled into the nutrition program. The community nutrition volunteers were trained and supported to include health education and promotion messages on immunization in addition to the routine nutrition education and counselling being provided. In addition, during mass screening for malnutrition using MUAC tape being done and Infant and Young Child Feeding (IYCF) counselling the immunization status of all children were assessed and appropriate vaccination provided by the vaccinators who was part of the team. The community nutrition volunteers tracked children who defaulted from both the nutrition and immunization programs using the facility records and child health cards. The defaulter tracking was incorporated as part of the community outreaches so any child who had missed any immunization session was appropriately vaccinated. The main hypothesis was that inclusion of immunization services during nutrition services both at the OTP and community outreaches be an effective strategy for reducing missed opportunity for immunizing all eligible children accessing nutrition services.

**Study site:** The study was conducted in Bentiu Protection of civilian (PoC) site in Rubkona County, Unity State established in December 2013. There were 112,140 IDPs and 20,219 households seeking protection in the site and is divided into 5 sectors (sector 1-5) [10]. There are 3 OTP centres (one in sector 2 and two in sector 5) and two Primary health care centres, one each in sector 2 and sector 5. Sector 2 had population of 20,315 while sector 5 had a total population of 33,379 individuals. The PHCC provide essential health care services including all routine immunization antigens in line with the government Expanded Program on Immunization (EPI) schedule while the OTP centres only provide nutrition services including community outreaches.

**Sampling:** Purposive sampling was used in the selection of the two OTP centres out of the 3 OTP centres in the camp used for the study. The two OTP centres (the only one in sector 2 and one out of the two in sector 5) were those the nutrition implementing partner agreed to integrate immunization into nutrition activities at OTP centres and community outreaches.

**Data collection and analysis:** All immunised children were routinely tallied and recorded in the national immunization registers to ensure accuracy during immunization sessions both in the OTP and during outreaches and all children had their vaccination status recorded in the child health card every time immunized which was used to determine the next vaccines and identify any child who had missed any immunization sessions during defaulter tracking in the community. The immunization coverage for various antigens were analysed and the immunization coverage among children receiving nutrition services both through the OTP and outreaches was compared with the immunization coverage for various antigens in the primary health care centres which routinely provide immunization services. Data were entered and analyzed using STATA version 15 and excel analysis software. The primary outcome measure was receipt of appropriate antigens by children assessing nutrition services and the secondary outcome measure was dropout in vaccination. Proportions were calculated for categorical variables and summarized into tables and figure for univariate analysis. Bivariate analysis using logistic regression was conducted to identify predictor of missed vaccinations. Odds Ratio (OR) and 95% Confidence Interval (95% CI) were used to estimate the strength of association between independent variables and the dependent variable. The threshold for statistical significance was set at p < 0.05. The dropout rate was calculated as a percentage of the difference between first and third pentavalent vaccine dose.

**Ethics approval and consent to participate:** The State Ministry of Health, Liech State reviewed and cleared the use of data, contents and publication of this manuscript.

## Results

**Figure 1:**
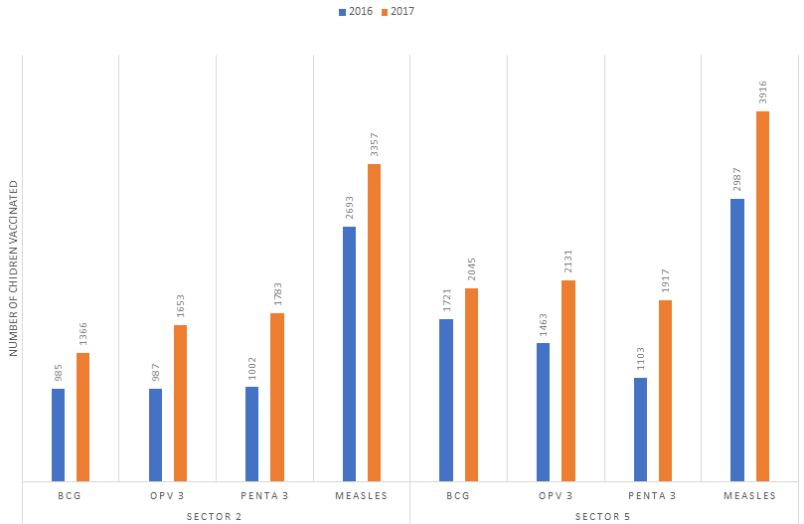
Children vaccinated in Bentiu POC sector 2 and sector 5 in 2016 compared with 2017

The comparison analysis of the immunization data showed that more children were immunized with various antigens in 2017 than in 2016. In sector 2 in 2016, the total number of children immunized with BCG vaccine was 985 compared to 1366 vaccinated with BCG vaccines in 2017, likewise the number of children who received third dose of Oral polio vaccine was 987 in 2016 compared with 1653 in 2017. The number of children vaccine with third dose of pentavalent vaccine and measles were 1002 and 2693 respectively in 2016 which were less than 1783 and 3357 children vaccinated with third dose of Penta valent vaccine and measles respectively in 2017. Similarly, in sector 5 in 2016, the total number of children immunized with BCG vaccine was 1721 compared to 2045 vaccinated with BCG vaccines in 2017, likewise the number of children who received third dose of Oral polio vaccine was 1463 in 2016 compared with 2131 in 2017. The number of children vaccinated with third dose of pentavalent vaccine and measles were 1103 and 2987 respectively in 2016 which were less than 1917 and 3916 children vaccinated with third dose of Penta valent vaccine and measles respectively in 2017 (Figure 1).

The analysis of the immunization data among malnourished children showed that a good number of malnourished children received all the appropriate immunization antigens needed. In the OTP centre in sector 2, a total of 1,646 malnourished children were enrolled into the nutrition program during the study period with 432(26.2%) of them immunised with BCG vaccines while 502(30.5%) received the third dose of Oral Polio Vaccines, 687(41.2%) were vaccinated with the third dose of pentavalent vaccines and 1482(90%) vaccinated with measles vaccines. In sector 5 OTP centre, a total of 2712 malnourished children were enrolled into the nutrition program during the study with 934(34.4%) of them immunised with BCG vaccines, 951(35.1%) received the third dose of Oral Polio Vaccine, 1096(40.4%) vaccinated with the third dose of pentavalent vaccines and 1875(69.1 %) were vaccinated with measles vaccines (Table 1). The analysis of the data on the total number of children immunized in the two study sites during the project period showed that integration of immunization into the nutrition services through the OTP centres and outreaches contributed immensely to the number of children immunized with various antigens. In Sector 2, through the OTP centre, 432(31.6%) of the total number of children immunized with BCG vaccine were reached while 502(34.5%) and 687(38.5%) of the total number children completed 3rd dose of Oral polio vaccine and Pentavalent vaccine respectively. A total of 1482 (44.2%) and 805 (50.7%) of children immunized in sector 2 with measles and injectable polio vaccine respectively were immunized through the OTP centre. In Sector 5, through the OTP centre, 419 (20.5%) of the total number of children immunized with BCG vaccine were reached while 453(23.5%) and 839(43.8%) of children completed 3^rd^ dose of Oral polio vaccine and Pentavalent vaccine respectively. A total of 1721(43.9%) and 743(30.3%) of children immunized in Sector 5 with measles and injectable polio vaccine respectively were immunized through the OTP centre (Table 2).

**Table 1:** Immunization coverage among children malnourished vaccinated at the OTP centers in sector 2 and sector 5

Antigen	Sector 2 N=1,646	Sector 5 N=2,712
	n (%)	n (%)
**BCG**	432(26.2)	934(34.4)
**OPV-0**	420(25.5)	823(30.3)
**OPV-1**	642(39.0)	1377(50.8)
**OPV-2**	559(34.0)	1042(38.4)
**OPV-3**	502(30.5)	951(35.1)
**Penta-1**	754(46.0)	1497(55.2)
**Penta-2**	713(43.3)	1255(46.2)
**Penta-3**	687(41.2)	1096(40.4)
**IPV**	830(50.4)	808(30.0)
**Measles**	1482(90.0)	1875(69.1)

**Table 2:** Immunization coverage among children vaccinated at the OTPs and PHCCs in sector 2 and sector 5

Antigen	SECTOR 2			SECTOR 5		
	OTP Centre	PHCC	Total	OTP centre	PHCC	Total
	n (%)	n (%)		n (%)	n (%)	
BCG	432(31.6)	934(68.4)	1366	419(20.5)	1626(79.5)	2045
OPV-0	420(33.8)	823(66.2)	1243	503(26.1)	1414(73.9)	1917
OPV-1	642(31.8)	1377(68.2)	2019	888(34.9)	1655(65.1)	2543
OPV-2	559(34.9)	1042(65.1)	1601	597(28.8)	1550(71.2)	2147
OPV-3	502(34.5)	951(65.5)	1453	453(23.5)	1478(76.5)	1931
Penta-1	754(33.5)	1497(66.5)	2251	954 (36.5)	1660(64.5)	2614
Penta-2	713(36.2)	1255(63.8)	1968	858(36.8)	1472(63.2)	2330
Penta-3	687(38.5)	1096(61.5)	1783	839(43.8)	1078(56.2)	1917
IPV	830(50.7)	808(49.3)	1638	743 (30.3)	1702(69.7)	2445
Measles	1482(44.2)	1875(55.8)	3357	1721(43.9)	2195(56.1)	3916

**Figure 2:**
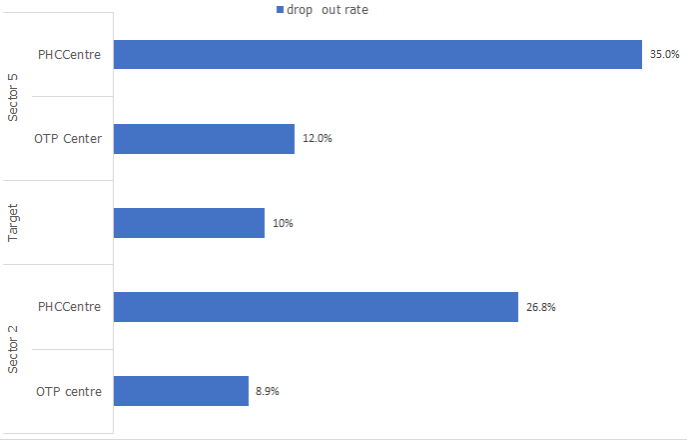
Dropout rate among children vaccinated in the OTP centres and PHC centres in Bentiu PoC in sector 2 and sector 5 in 2017

The analysis of the dropout rate which was defined as the percentage difference between children who received 3^rd^ dose of pentavalent vaccine ( Penta 3) and those who received 1st dose of the pentavalent vaccine(Penta 1) showed that in sector 2, among those children who received the first dose of pentavalent vaccine through the OTP centre, 8.9% did not return for their third dose of pentavalent vaccine while 26.8% of children who received the first does of pentavalent vaccine through the PHCC did not return for the third dose of pentavalent vaccines. In sector 5, among those children who received the first dose of pentavalent vaccine through the OTP centre, 12% did not return for their third dose of pentavalent vaccine while 35 % of children who received the first does of pentavalent vaccine through the PHCC did not return for the third dose of pentavalent vaccines. The acceptable limit is 10% (Figure 2). On bivariate analysis using logistic regression to identify the predictor of missed vaccination, children who were vaccinated at the OTP centre in sector 2 were 45% less likely to miss vaccination than those vaccinated at the PHCC (OR: 0.45; 95%CI: 0.36- 0.55 and was found to be statistically significant (p<0.05) while those vaccinated at the OTP sector in sector 5 were 27% less likely to miss vaccination than those vaccinated at the PHCC (OR: 0.27; 95%CI: 0.20 -0.35) also found to be statistically significant (p<0.05 (Table 3).

**Table 3:** Bivariate analysis of factors associated with missed vaccination among children attending immunization services in Bentiu PoC

Variables	Drop out n (%)	No drop out n (%)	Odd Ratio (95% Confidence Interval)
**Sector 2**			
OTP Centre	115(12)	839(88)	0.45 (0.36 -0.55)
PHC Centre	582(35)	1917(65)	P<0.05
**Sector 5**			
OTP Centre	67(8.9)	687(91.1)	0.27 (0.20-0.35)
PHC Centre	401(27)	1096(73)	P<0.05

## Discussion

In most studies on integration [8, 11], immunization program was used as platform for integrating other MNCH services unlike this study where nutrition program was used as the entry point/ platform for integration of immunization services. Most studies [12-14] where integration was done using nutrition program as platform, were mostly co-location than co-delivery where children who came for nutrition services were referred to other immunization services delivery points unlike this study where both immunization and nutrition services were co-delivered both at the OTP centres and during outreaches. In this study there was increase in the number of children vaccinated in the project sites during the project year compared to the previous year. The malnourished children attending the OTP centres were provided with appropriate immunization antigens which they could have missed if the services were not provided because of the expanded packages of services provided at the OTP centres and this intervention could have contributed to the nutrition outcome reported with cure rate of more than 80% in the two OTP sites. In this study the number of children immunized through the integration in the nutrition services contributed greatly to the coverage of all immunization antigens in the project sites and so reduced greatly the missed opportunity for immunization services among the vulnerable population this is similar to findings from two studies [15, 16] where nutrition was used as platform for integration on immunization services. In these two related studies [15, 16] on Growth Monitoring Program Plus programme, which involved providing monthly outreach services implemented in peri-urban areas of Lusaka, Zambia immunization coverage was shown to significantly increase. In a randomised controlled intervention trial [14] conducted in seven Special Supplementary Program for Women, Infant and Children sites (WICs) in Chicago, in the intervention sites immunization activities were included in the routine nutrition activities which involved screening for all children under 5 during every visit to the WIC, referral of every eligible children for immunization and use of food vouchers for mothers to bring their children for immunization when needed, the vaccination coverage was reported significantly increased in the intervention group. However, the study showed that that conducting assessment and referral of women, infant and children in the nutrition program to immunization points without other accompanying interventions did not raise vaccination coverage and showed no evidence that immunization assessment and referral alone increased immunization coverage in this population and concluded that the use of voucher incentives has the strongest evidence of effectiveness in increasing immunization rates. This is the belief in this study that the plumpy nuts provided would have been a form of incentives to care givers and mothers to bring their children for the services.

In this study, the dropout rate was much lower and statistically significant among children who received immunization at the OTP centres than those in the PHC Centre, this may be attributed to the incentives received at the clinic in term of therapeutic food by the child parents and the tracking of defaulter integrated with the tracking of OTP patients, this is similar to a randomised controlled intervention trial [15] conducted in seven Special Supplementary Program for Women, Infant and Children sites (WICs) in Chicago which reported significant lower dropout rate in the intervention groups with the use of voucher incentives being the motivation/incentive for completeness of immunization. The role of incentives in reducing drop out was reported in a randomised study conducted in India [17] which showed significant reduction in immunization dropout rate when mothers and care givers were provided incentive, the plumpy nuts being provided at the OTP centres could actually be a form of incentives which caregivers/mothers perceived as an immediate need unlike vaccination which they possibly perceived as less urgent for mothers/care givers to be explored to complete immunization and reduce drop out. This study demonstrated that program with better funding can be used as the platform for integrating other services, here fund from different donors were pooled together to provide the two services with significant impact on the children in the communities, not only the children vaccinated but will ensure adequate coverage to improve on the herd immunity and prevent outbreak of disease especially measles and its complications. The vaccinators were paid with other grants while the nutrition intervention and transport means funded by nutrition donor as an example of efficient and effective use of health sector resources. This is similar to the project cited in other study which demonstrated efficient co-sharing/pooling of resources from donor funded vertical health programs where the better-funded malaria control programs were used as platform for integrating immunization, vitamin A distribution, and deworming outreach sessions [18].

Integration has evolved to be one of the service delivery models with the potential to enhance improve coverage, efficiencies, synergies and child survival outcomes [19]. Yet, understanding and implementing effective models of integrated approaches remain difficult. This initiative provided a unique opportunity to expand the knowledge of integration to facilitate and institutionalise integrated delivery of immunization and nutrition services which are traditionally vertical programs especially in South Sudan The planning, implementation and results of this integration of immunization into OTP centres in humanitarian setting with limited funding opportunities highlights a few critical issues that are important for future scale up of such integration and adopted as a national strategy. Integrating routine immunization into nutrition services if well planned and organized, is feasible and acceptable with little or no negative consequences on the both services. The integration in this study is a model of co-delivery of services unlike some form of co-location of services a model of integration that other health programs such as HIV and Tuberculosis (TB) have implemented for decades [20] but have failed to adequately institutionalize their integration and *co-delivery* of services in one encounter [21].

## Conclusion

This study indicated that retention rates and completed vaccination improved effectively with integration with nutrition services as a model of an integrated immunization programme for child health in line with the Integrated Management of Childhood Illnesses (IMCI) and the Global Immunization Vision and Strategy (GIV) and with adequate planning, integration can improve coverage, combine costs and create synergies. In order to deliver on the promise of Sustainable Development Goals 2 and 3 and dramatically reduce child mortality by 2030 we must ensure that every child has access to life-changing vaccines that not only provide protection against the leading causes of death in children under five, but also contribute to decreasing vulnerability to malnutrition.

**Limitation of the study:** this study is based on review of clinic records and subject to the limitations associated with studies utilizing routine data. In addition, the study did not evaluate the possible impact of immunization status on the nutrition outcome among malnourished children who completed their immunization and those who dropped out.

### What is known about this topic

- A child who is borderline nourished will tip into malnutrition if he or she contracts an infectious disease such as measles. As part of an integrated health approach, immunization plays a critical role in curbing the devastating impact of malnutrition;
- Immunization and Nutrition services are mostly vertical program in most countries.

### What this study adds

- The number of children immunized through the integration in the nutrition services contributed greatly to the coverage of all immunization antigens in the project sites;
- Integration reduced greatly the missed opportunity for immunization services among the vulnerable population.

## Competing interests

The authors declare no competing interests.
